# Identifying Functional Status Impairment in People Living With Dementia Through Natural Language Processing of Clinical Documents: Cross-Sectional Study

**DOI:** 10.2196/47739

**Published:** 2024-02-13

**Authors:** John Laurentiev, Dae Hyun Kim, Mufaddal Mahesri, Kuan-Yuan Wang, Lily G Bessette, Cassandra York, Heidi Zakoul, Su Been Lee, Li Zhou, Kueiyu Joshua Lin

**Affiliations:** 1 Department of Medicine Brigham and Women's Hospital Boston, MA United States; 2 Harvard Medical School Boston, MA United States; 3 Marcus Institute for Aging Research Hebrew SeniorLife Boston, MA United States; 4 National Cheng Kung University Hospital Tainan Taiwan; 5 Massachusetts General Hospital Boston, MA United States

**Keywords:** activities of daily living, ADLs, clinical note, dementia, electronic health record, EHR, functional impairment, instrumental activities of daily living, iADLs, machine learning, natural language processing, NLP

## Abstract

**Background:**

Assessment of activities of daily living (ADLs) and instrumental ADLs (iADLs) is key to determining the severity of dementia and care needs among older adults. However, such information is often only documented in free-text clinical notes within the electronic health record and can be challenging to find.

**Objective:**

This study aims to develop and validate machine learning models to determine the status of ADL and iADL impairments based on clinical notes.

**Methods:**

This cross-sectional study leveraged electronic health record clinical notes from Mass General Brigham’s Research Patient Data Repository linked with Medicare fee-for-service claims data from 2007 to 2017 to identify individuals aged 65 years or older with at least 1 diagnosis of dementia. Notes for encounters both 180 days before and after the first date of dementia diagnosis were randomly sampled. Models were trained and validated using note sentences filtered by expert-curated keywords (filtered cohort) and further evaluated using unfiltered sentences (unfiltered cohort). The model’s performance was compared using area under the receiver operating characteristic curve and area under the precision-recall curve (AUPRC).

**Results:**

The study included 10,000 key-term–filtered sentences representing 441 people (n=283, 64.2% women; mean age 82.7, SD 7.9 years) and 1000 unfiltered sentences representing 80 people (n=56, 70% women; mean age 82.8, SD 7.5 years). Area under the receiver operating characteristic curve was high for the best-performing ADL and iADL models on both cohorts (>0.97). For ADL impairment identification, the random forest model achieved the best AUPRC (0.89, 95% CI 0.86-0.91) on the filtered cohort; the support vector machine model achieved the highest AUPRC (0.82, 95% CI 0.75-0.89) for the unfiltered cohort. For iADL impairment, the Bio+Clinical bidirectional encoder representations from transformers (BERT) model had the highest AUPRC (filtered: 0.76, 95% CI 0.68-0.82; unfiltered: 0.58, 95% CI 0.001-1.0). Compared with a keyword-search approach on the unfiltered cohort, machine learning reduced false-positive rates from 4.5% to 0.2% for ADL and 1.8% to 0.1% for iADL.

**Conclusions:**

In this study, we demonstrated the ability of machine learning models to accurately identify ADL and iADL impairment based on free-text clinical notes, which could be useful in determining the severity of dementia.

## Introduction

In the United States, over 6 million people are living with Alzheimer disease or related dementia, and this number is projected to increase to 13 million by 2050 [1]. As dementia progresses, the affected individuals lose the ability to carry out everyday activities, including basic activities of daily living (ADLs) and instrumental ADLs (iADLs), which are fundamental skills required to independently care for oneself and serve as an indicator of a person’s functional status [2]. Assessment of ADLs and iADLs is also essential in determining the severity of dementia and the care needs of older adults [3]. This information is important in predicting a patient’s risk of mortality, long-term nursing home admission, and health care use.

Despite the significance of assessing disability in performing ADLs and iADLs in people living with dementia, the assessment is not routinely done in clinical practice; if performed, it is often documented within the unstructured clinical notes of a patient’s electronic health record (EHR), making it difficult to readily locate. This process could be improved with natural language processing (NLP) and machine learning. NLP has been applied to health care research in a variety of ways, including quantifying changes in social media posts, better understanding the mental health impacts of COVID-19 [4], and extracting cancer phenotypes from clinical note text [5]. An NLP approach can convert free-text information on ADLs and iADLs from an EHR into structured data. The structured data of ADL and iADL can then be readily used in clinical care and research to perform statistical modeling for outcome prediction, patient phenotyping, and confounding or risk adjustment. In this study, we seek to develop and validate machine learning models that can identify clinical note text containing information on ADL and iADL impairment in people living with dementia.

## Methods

### Setting and Data Sources

We used data from the Research Patient Data Repository (RPDR) [6] linked to Medicare fee-for-service parts A (inpatient coverage), B (outpatient coverage), and D (prescription benefits) claims data for over 700,000 individuals from 2007 to 2017. The RPDR includes longitudinal EHR data from 2 tertiary hospitals, 3 community hospitals, and more than 35 primary care centers in Greater Boston, Massachusetts. This data set includes demographic information, inpatient and outpatient diagnoses and procedures, medical orders and drug prescriptions, vital signs, laboratory and radiology test results, and free-text notes and reports from inpatient and ambulatory encounters. We linked the EHR with Medicare claims data to reduce information leakage of the EHR due to care provided outside of our EHR [7,8]. The linkage was done deterministically by the unique Medicare beneficiary number, date of birth, and sex, with a success linkage rate of 98.7% [9]. Medicare is a US federal health insurance program that currently covers approximately 50 million Americans by providing medical and prescription drug coverage to individuals aged 65 years or older and to younger individuals with disabilities. The Medicare claims database contains longitudinal, individual-level data on health care use, diagnoses, diagnostic tests, procedures, and pharmacy-filled prescriptions.

### Study Population

From the linked RPDR-claims data from 2007 to 2017, we identified older adults aged 65 years or older with at least 1 diagnosis of dementia using validated algorithms with positive predictive values of 78%-92% to define dementia [10]. The first date of dementia diagnosis during our study period was the cohort entry (index) date. We further required the study cohort to have at least 365 days of continuous enrollment in Medicare parts A and B and at least 1 admission, progress, or discharge note from an inpatient encounter or ambulatory visit within 365 days before the index date.

### Labeled Data Set Development

Patient clinical notes were split into sentences using the Medical Text Extraction, Reasoning, and Mapping System NLP system [11]. In our preliminary exploration, the information relevant for ADL and iADL was noted to be sparse, resulting in a highly imbalanced data set with little input data for model development. To increase the prevalence of data containing information relevant to ADL and iADL impairment in our model training data set, we created a lexicon of ADL- and iADL-related key terms curated with physician expert guidance. A total of 3 medical doctors (DHK, MM, and KJL) came up with the initial list of terms with automatically generated synonyms, followed by list refinement based on clinical knowledge (see Table S1 in Multimedia Appendix 1 for a final list of the terms). We filtered and kept sentences that included at least 1 key term from the final lexicon using a filtering tool coded in Python. Notes were split into sentences but retained a context window of 250 characters before and after key terms. Each medical doctor was paired with a research assistant to form a review team. The 3 review teams first conducted preliminary reviews on the same set of notes, filtered by our key term lexicon. They discussed sentences with different classifications regarding ADL and iADL impairment and assessed interrater agreement (κ was 85.2%, 89.8%, and 85.4% between the 3 teams after 5 rounds of training sessions). Then the review teams manually labeled 10,000 randomly selected filtered sentences in 2743 clinical notes from 441 patients for evidence of ADL or iADL impairment. The data were randomly split into a 70% subset (filtered training cohort) and a 30% subset (filtered validation cohort). We used the training cohort to train and tune model parameters by 5-fold cross-validation and used the validation cohort to test the performance. Models were further evaluated using 1000 randomly selected, unfiltered sentences from an independent set of 80 patients for assessing generalizability.

### Classifier Development

We implemented 5 commonly used statistical models in machine learning literature: logistic regression, support vector machine (SVM), least absolute shrinkage and selection operator (LASSO) regression [12], random forest [13], implemented using the Python Scikit-learn module [14], and gradient boosting, implemented using the Python XGBoost module [15]. Training data for these 5 models were represented as unigrams transformed using term frequency—inverse document frequency [16]. We also implemented a hierarchical attention-based deep learning model consisting of a convolutional neural network and long short-term memory network, developed in a previous study [17]. Additionally, we implemented a model derived from Bio+Clinical bidirectional encoder representations from transformers (BERT), a contextualized word representation model based on BioBERT, and trained further on Medical Information Mart for Intensive Care (MIMIC) data [18-21]. We performed 5-fold cross-validation on the training cohort to tune the parameters for each model based on area under the receiver operating characteristic curve (AUROC) and area under the precision-recall curve (AUPRC) metrics. Tuned model performance was then evaluated on the filtered validation cohort, and generalizability was tested on the unfiltered validation cohort (ie, the 1000 unfiltered sentence set). See Tables S2 and S3 in Multimedia Appendix 1 for the final parameters of the ADL and iADL impairment classifiers, respectively.

### Ethical Considerations

The study was approved by the institutional review board of Brigham and Women’s Hospital, Boston, Massachusetts (2018P002462). Personal health information was used as minimally as possible within the needs of the study. Data were not shared with any individuals not directly involved in the study.

## Results

### Study Sample

The filtered cohort was used to extract our data set of 10,000 filtered sentences; it comprises 441 people with a mean age of 82.7 (SD 7.9) years. A total of 64% (n=283) of the people were female, 88% (n=389) were White, and 4% (n=19) were Black. During the 365-day baseline period, the mean frailty score in our cohort was 0.26 (SD 0.08) with the most commonly observed comorbidities being urinary tract infections (n=192, 43.5%), history of falls (n=172, 39%), failure to thrive (n=89, 20.2%), incontinence (n=73, 16.5%), pressure ulcers (n=57, 12.9%), and dysphagia (n=50, 11.3%). As far as health care use is concerned, people on average had 13 (SD 7.8) unique medications along with a mean of 11.5 (SD 8.8) outpatient visits in the baseline period. Furthermore, the mean number of baseline hospitalizations and emergency room visits were 1.3 (SD 1.7) and 2.4 (SD 2.7), respectively. Our key-term filtered data set contained 1628 (16.3%) sentences annotated as positive (ie, containing relevant information) for ADL impairment (1128 in the training subset and 500 in the internal evaluation subset) and 323 (3.2%) sentences annotated as positive for iADL impairment (234 in the training subset and 89 in the internal evaluation subset). In contrast, the unfiltered data set was used to extract the external validation cohort of 1000 unfiltered sentences from 80 patients that contained 7 (0.7%) sentences labeled positive for ADL impairment and 4 (0.4%) sentences labeled positive for iADL impairment. Compared to the filtered data set, the unfiltered data set has a comparable mean age and race composition but a slightly higher female percentage (56/80, 70% vs 283/441, 64.2%). The baseline comorbidity profile of the 2 study cohorts was largely comparable, except that the prevalence of aspiration pneumonia was higher in the filtered than unfiltered set. The health care use was also noted to be slightly higher in the filtered than in the unfiltered set (Table 1).

**Table 1:** Selected characteristics of the filtered and unfiltered study data set.

Covariate			Filtered 10,000-sentence sample (n=441)		Unfiltered 1000-sentence sample (n=80)	Absolute standardized difference
Age (years), mean (SD)			82.7 (7.9)		82.8 (7.5)	0.02
**Age categories (years), n (%)**						
	65-70	36 (8.2)		5 (6)		0.07
	71-75	51 (11.6)		9 (11)		0.01
	76-80	68 (15.4)		12 (15)		0.01
	81-85	96 (21.8)		17 (21)		0.01
	>85	190 (43.1)		37 (46)		0.06
**Sex, n (%)**						
	Female	283 (64.2)		56 (70)		0.12
	Male	158 (35.9)		24 (30)		0.12
**Race, n (%)**						
	White	389 (88.2)		70 (88)		0.02
	Black	19 (4.3)		4 (5)		0.03
	Other	33 (7.5)		6 (8)		0.00
Frailty score, mean (SD)			0.26 (0.1)		0.25 (0.1)	0.06
**Comorbidities, n (%)**						
	Falls	172 (39.0)		30 (38)		0.03
	Pressure ulcer	57 (12.9)		11 (14)		0.02
	Failure to thrive or body weight loss	89 (20.2)		15 (19)		0.04
	Use of feeding tube	7 (1.6)		2 (3)		0.06
	Aspiration pneumonia	33 (7.5)		1 (1)		0.31
	UTI^a^	192 (43.6)		34 (43)		0.02
	Incontinence	73 (16.6)		14 (18)		0.03
	Dysphagia (eating problems)	50 (11.3)		6 (8)		0.13
**Medications, n (%)**						
	1st line dementia medication use	49 (11.1)		7 (9)		0.08
	Memantine use	21 (4.8)		4 (5)		0.01
	Antipsychotic medication use	38 (8.7)		8 (10)		0.05
**Health care use, mean (SD)**						
	Hospitalizations, n	1.32 (1.7)		1.25 (2)		0.04
	ER^b^ visits, n	2.40 (2.7)		2.00 (2)		0.16
	Outpatient visits, n	11.54 (8.8)		10.15 (9)		0.16
	Medications, n	13.06 (7.8)		11.94 (8)		0.14
Nursing home stay of >100 days in baseline, n (%)			17 (3.9)		6 (8)	0.16
Hospice care, n (%)			12 (2.7)		4 (5)	0.12

^a^UTI: urinary tract infection.

^b^ER: emergency room.

### Performance of the ADL Model

**Figure 1:**
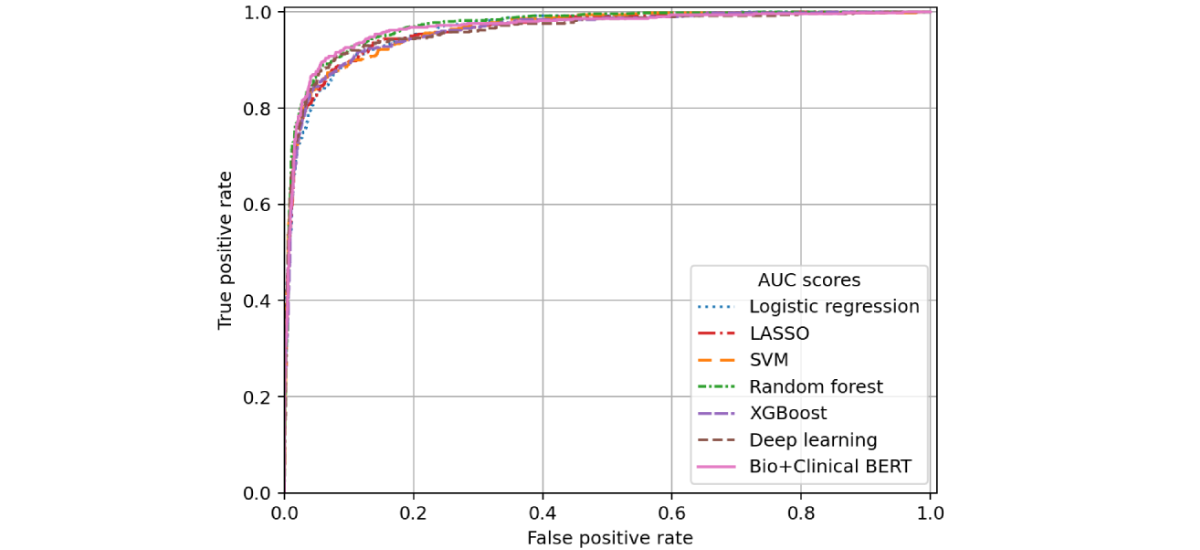
Receiver operating characteristic curves for activity of daily living impairment prediction performance on the filtered validation subset. AUC: area under the curve; BERT: bidirectional encoder representations from transformers; LASSO: least absolute shrinkage and selection operator; SVM: support vector machine.

**Figure 2:**
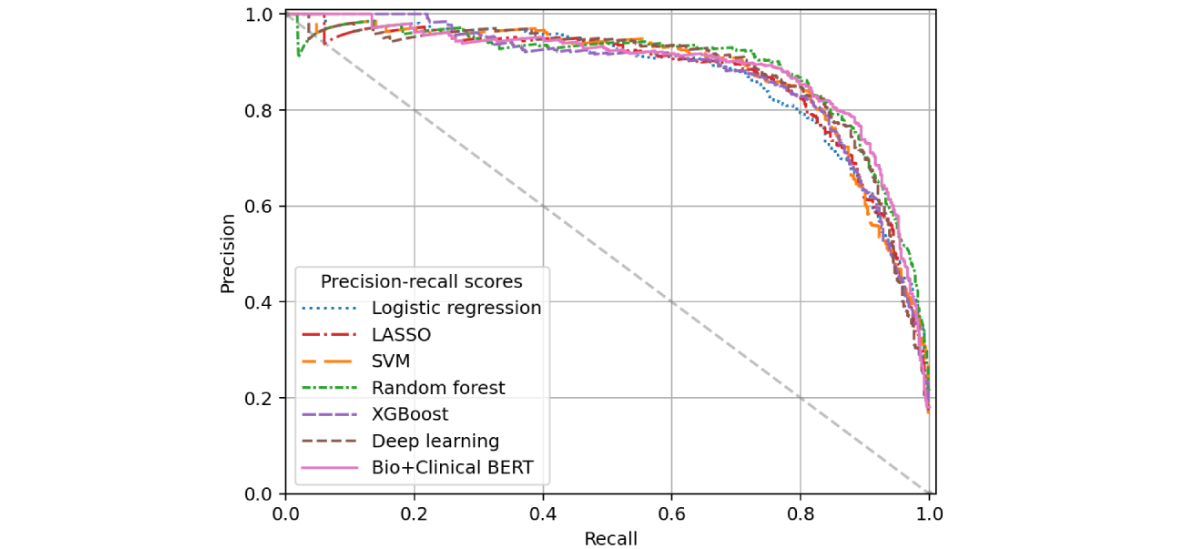
Precision-recall curves for activity of daily living impairment prediction performance on the filtered validation subset. BERT: bidirectional encoder representations from transformers; LASSO: least absolute shrinkage and selection operator; SVM: support vector machine.

AUROC and AUPRC performance across all models for ADL impairment detection in the training set and both evaluation sets are shown in Table 2 with receiver operating characteristic (ROC) and precision-recall curves for the filtered validation cohort evaluation in Figures 1 and 2, respectively. While most models scored high AUROC across data sets, there was more notable variation in AUPRC scores, particularly in the unfiltered validation set. LASSO performed best in training set cross-validation, with an AUROC of 0.958 and AUPRC of 0.865. Top predictors of the LASSO model include “incontinence,” “tube,” “incontinent,” “PEG (percutaneous endoscopic gastrostomy),” “bathing,” “dressing,” “feeding,” “total parenteral nutrition (TPN),” “assistance,” and “toileting.” These features tended to have high importance for the remaining models, as well as “gastrostomy” and “body.” As shown in Table 2, most ADL models performed similarly on the filtered validation cohort, with the random forest model achieving slightly better AUROC and AUPRC measures (0.971 and 0.887, respectively) than the others. All models’ AUROC scores improved for the unfiltered validation cohort, explained by the notable imbalance of the data set—only 0.7% (7/1000) cases were positive for ADL impairment in the unfiltered validation cohort versus 16.7% (500/3000) in the filtered validation cohort. Unfiltered validation AUROC was highest for the deep learning model (0.991). The AUPRC scores of all models decreased for the unfiltered validation cohort prediction, with the SVM model’s score the highest (0.822) and dropping the least. Though trained on more data than the deep learning model, the data that the Bio+Clinical BERT model is pretrained on is not specific to the Mass General Brigham (MGB) EHR. This is likely why Bio+Clinical BERT exhibited lower performance than the deep learning model, which was trained entirely on our annotated MGB data set.

**Table 2:** Activities of daily living classifier performance. Italic values represent the optimal performance in each data set.

Model			AUROC^a^ (95% CI)	AUPRC^b^ (95% CI)
**Filtered cohort**				
	**Training set**			
		Deep learning	0.952 (0.945-0.960)	0.864 (0.844-0.881)
		Bio+Clinical BERT^c^	0.870 (0.842-0.897)	0.826 (0.789-0.862)
		Logistic regression	0.955 (0.949-0.962)	0.855 (0.837-0.872)
		LASSO^d^	*0.958 (0.952-0.965)*	*0.865 (0.846-0.883)*
		Random forest	0.953 (0.946-0.960)	0.857 (0.838-0.875)
		SVM^e^	0.954 (0.946-0.960)	0.854 (0.835-0.872)
		XGBoost	0.955 (0.948-0.962)	0.848 (0.826-0.869)
	**Validation set**			
		Deep learning	0.961 (0.951-0.971)	0.880 (0.852-0.906)
		Bio+Clinical BERT	0.873 (0.852-0.891)	0.847 (0.823-0.869)
		Logistic regression	0.963 (0.954-0.971)	0.871 (0.841-0.896)
		LASSO	0.962 (0.954-0.970)	0.870 (0.841-0.896)
		Random forest	*0.971 (0.964-0.977)*	*0.887 (0.859-0.913)*
		SVM	0.963 (0.954-0.971)	0.877 (0.849-0.902)
		XGBoost	0.961 (0.951-0.969)	0.873 (0.846-0.898)
**Unfiltered validation cohort**				
	Deep learning		*0.991 (0.986-0.994)*	0.817 (0.746-0.882)
	Bio+Clinical BERT		0.785 (0.582-0.999)	0.621 (0.227-0.901)
	Logistic regression		0.981 (0.971-0.990)	0.737 (0.644-0.817)
	LASSO		0.969 (0.954-0.983)	0.675 (0.573-0.769)
	Random forest		0.990 (0.984-0.995)	0.806 (0.723-0.880)
	SVM		0.986 (0.975-0.994)	*0.822 (0.748-0.887)*
	XGBoost		0.978 (0.959-0.992)	0.771 (0.680-0.846)

^a^AUROC: area under the receiver operating characteristic curve.

^b^AUPRC: area under the precision-recall curve.

^c^BERT: bidirectional encoder representations from transformers.

^d^LASSO: least absolute shrinkage and selection operator.

^e^SVM: support vector machine.

### Performance of the iADL Model

**Figure 3:**
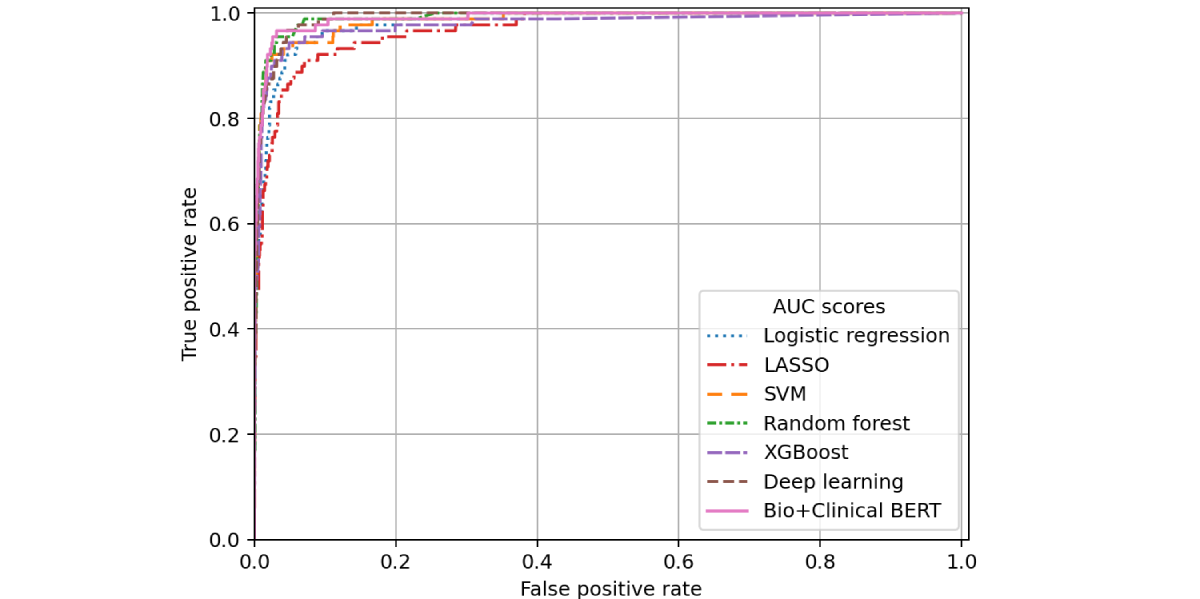
Receiver operating characteristic curves for instrumental activity of daily living impairment prediction performance on the filtered validation subset. AUC: area under the curve; BERT: bidirectional encoder representations from transformers; LASSO: least absolute shrinkage and selection operator; SVM: support vector machine.

**Figure 4:**
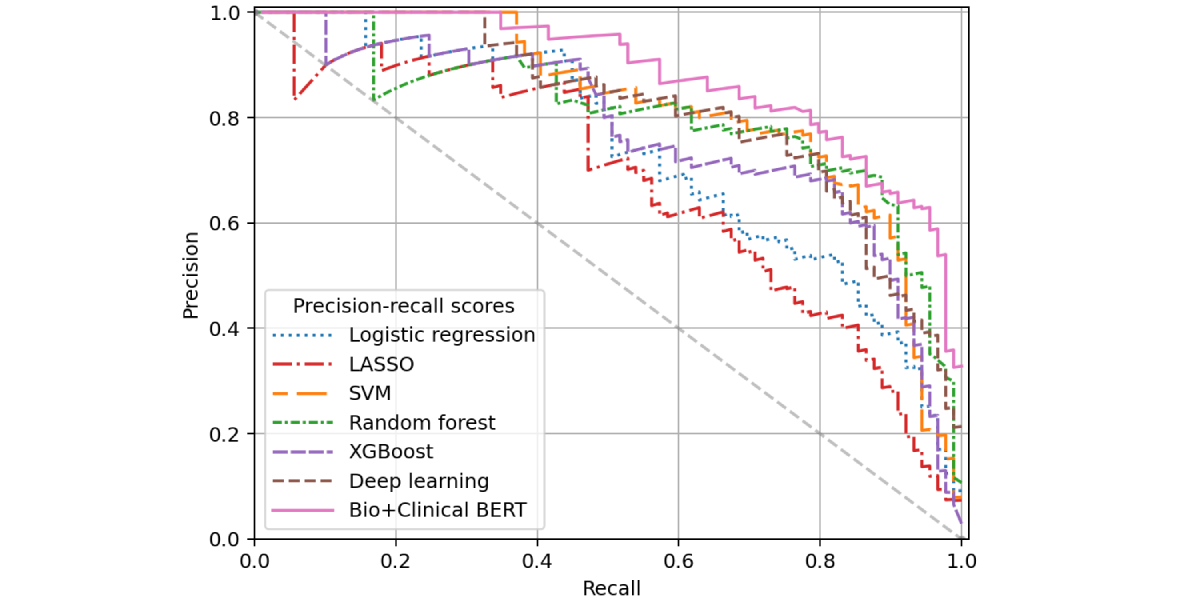
Precision-recall curves for instrumental activity of daily living impairment prediction performance on the filtered validation subset. BERT: bidirectional encoder representations from transformers; LASSO: least absolute shrinkage and selection operator; SVM: support vector machine.

Evaluation data sets were more imbalanced for the iADL impairment classification task—the filtered validation cohort had a 3.0% positive rate for iADL impairment and just 0.4% in the unfiltered validation cohort. This resulted in wide CIs for reported performance. Across data sets, AUROC scores remained high for all models except the deep learning and Bio+Clinical BERT models, which may have been hindered due to the low prevalence of positive training instances (n=234, 3.3% of the training set). Table 3 presents model performance results for iADL impairment detection in the training and 2 evaluation sets. Logistic regression and XGBoost performed best in training set cross-validation AUROC (0.97), while SVM produced the highest AUPRC (0.735). Top predictors of the LASSO model include iADL-related terms such as “cooking,” “shopping,” “management,” “laundry,” “finances,” “meals,” “cleaning,” “food,” and “medication.” These features also tend to have high importance for the remaining models, along with “husband,” “drives,” and “bills.” The XGBoost model’s AUROC scores were best for both data sets (0.995 for filtered validation and 0.991 for unfiltered validation), while the Bio+Clinical BERT model had the highest AUPRC scores for each validation data set (0.551 filtered and 0.568 unfiltered). ROC curves for the filtered validation cohort iADL classification appear in Figure 3, and precision-recall curves are provided in Figure 4.

**Table 3:** Instrumental activities of daily living classifier performance. Italic values represent the optimal performance in each data set.

Model			AUROC^a^ (95% CI)	AUPRC^b^ (95% CI)
**Filtered cohort**				
	**Training set**			
		Deep learning	0.948 (0.931-0.964)	0.677 (0.617-0.736)
		Bio+Clinical BERT^c^	0.860 (0.797-0.918)	0.730 (0.625-0.826)
		Logistic regression	*0.970 (0.958-0.980)*	0.714 (0.656-0.766)
		LASSO^d^	0.961 (0.945-0.975)	0.704 (0.644-0.758)
		Random forest	0.966 (0.951-0.979)	0.722 (0.668-0.774)
		SVM^e^	0.968 (0.955-0.980)	*0.735 (0.679-0.786)*
		XGBoost	*0.970 (0.956-0.981)*	0.703 (0.644-0.765)
	**Validation set**			
		Deep learning	0.806 (0.243-1.00)	0.551 (0.003-1.00)
		Bio+Clinical BERT	0.830 (0.777-0.876)	*0.758 (0.679-0.818)*
		Logistic regression	0.952 (0.901-0.998)	0.396 (0.067-0.803)
		LASSO	0.978 (0.935-0.999)	0.414 (0.155-0.869)
		Random forest	0.941 (0.863-0.998)	0.309 (0.062-0.744)
		SVM	0.934 (0.792-0.998)	0.430 (0.125-0.831)
		XGBoost	*0.995 (0.988-0.999)*	0.528 (0.255-0.925)
**Unfiltered validation cohort**				
	Deep learning		0.794 (0.191-1.00)	0.568 (0.002-1.00)
	Bio+Clinical BERT		0.750 (0.499-1.00)	*0.584 (0.001-1.00)*
	Logistic regression		0.960 (0.869-1.00)	0.538 (0.014-1.00)
	LASSO		0.986 (0.972-0.999)	0.271 (0.042-0.833)
	Random forest		0.945 (0.828-1.00)	0.521 (0.011-1.00)
	SVM		0.959 (0.867-1.00)	0.456 (0.022-1.00)
	XGBoost		*0.991 (0.972-1.00)*	0.552 (0.050-1.00)

^a^AUROC: area under the receiver operating characteristic curve.

^b^AUPRC: area under the precision-recall curve.

^c^BERT: bidirectional encoder representations from transformers.

^d^LASSO: least absolute shrinkage and selection operator.

^e^SVM: support vector machine.

### Clinical Implications

Table S4 in Multimedia Appendix 1 provides examples identified by our NLP classifier of various ways that ADL and iADL impairment can present in patient note text. The most prevalent key term categories (Table S1 in Multimedia Appendix 1) across the unfiltered cohort data set appear in Tables S5 and S6 in Multimedia Appendix 1 for ADL and iADL impairment, respectively. As shown by these tables, common ADL- and iADL-related terms appear in just a small fraction of patient note sentences. Using NLP can demonstrably improve the ability to locate evidence of diverse ADL and iADL impairments within patient notes. We performed a key term search on the unfiltered cohort data set using our ADL and iADL lexicons to see how well such an approach identified patient note sentences with evidence of ADL and iADL impairment. Key term search proved to be a sensitive approach—no false negatives occurred for ADL impairment identification and 1 (0.1%) occurred for iADL impairment identification. The false positive rate, however, was greater (45/1000, 4.5% for ADL vs 18/1000, 1.8% for iADL) when compared with the highest performing machine learning models (2/1000, 0.2% for ADL vs 1/1000, 0.1% for iADL).

## Discussion

### Principal Findings

Among people living with dementia in a cohort using US multicenter EHRs linked with Medicare claims data, we developed and validated NLP models to determine evidence of ADL and iADL impairment. Although the proportion of sentences in clinical notes that contained ADL and iADL information was low, our best-performing models effectively identified relevant sentences, with an AUROC of 0.990 (95% CI 0.984-0.995) for ADL (random forest) and 0.991 (95% CI 0.972-1.00) for iADL (XGBoost).

Identifying people living with dementia who have difficulty with basic ADLs and iADLs is important for clinical care and population health management. The degree of ADL and iADL impairment is associated with dementia severity and progression. The iADLs begin to decline at the mild cognitive impairment stage [22]. Widely used dementia severity scales, such as the Clinical Dementia Rating and Functional Assessment Staging Tool, require assessment of iADLs and ADLs. As the ability to perform iADLs and ADLs declines with the progression of dementia [23], early detection of iADL and ADL impairment can lead to early rehabilitation to preserve their daily function. In acute hospital care settings, assessment of iADL and ADL function could help identify those at risk of loss of independence and arrange care transition interventions [24]. Moreover, ADL dependence is a risk factor for falls in community-dwelling adults with dementia [25]. Similarly, iADL impairment is predictive of 30-day readmission and can be helpful in identifying high-risk patients for early interventions [26].

Despite the importance of ADL and iADL assessment, documentation of this information is neither standardized nor available in most EHR and claim data. As a result, measures of ADL and iADL impairment are not included in prediction models of readmission. Recently, an effort to use machine learning methods to extract ADLs and iADLs information from EHR free-text notes or reports showed a potential to improve risk prediction or clinical decision support systems. The iADL impairment identified using machine learning was predictive of 30-day readmission [26]. Similarly, geriatric syndromes that are not documented in structured EHR data can be further identified in unstructured clinical notes in the EHR using NLP algorithms [27]. It has also been shown that frailty described in clinical notes was associated with greater health care use [28].

### Comparison to Previous Work

Our work adds to previous research by showing the utility of NLP and machine learning algorithms to identify ADL and iADL information in unstructured EHR data with high accuracy for older adults with dementia. ADL and iADL impairment information from clinical notes of people living with dementia can help researchers identify medically stable and ambulatory older adults with dementia and specific functional levels who can be enrolled in clinical trials. In addition, information on ADL and iADL function is an important confounder in administrative claims-based studies of medical interventions in the fields of geriatrics, neurology, rehabilitation medicine, and family medicine. Combining our NLP approach with other data from the EHR could further improve the validity of EHR-based analysis. The extent of confounding and further adjustment has become possible through EHR-claims linkage in clinical research networks [8,29].

### Study Limitations

Our study has several limitations. Our model is based on a US metropolitan academic care delivery network. Because of the subjectivity of self-reported information, variations in documentation conventions, and different demographic or cultural backgrounds of the study population, it is unclear if our findings can be generalizable to other health care systems. Additionally, our findings were based on a relatively small cohort with 441 patients in the filtered set and 80 in the unfiltered set, so validation in a larger cohort, preferably with a different demographic profile, is needed to confirm generalizability. Additionally, the measure and conceptualization of iADLs can be complex due to the differences between cultural norms and gender roles. For example, women have greater health-related iADL limitations than men [30]. Cross-national variations in ADL and iADL impairment may reflect item-response bias due to culture-based gender norms rather than actual differences in disability levels [31].

### Conclusion

In conclusion, we have developed models to determine ADL and iADL impairment among US Medicare beneficiaries using NLP and EHR unstructured data. Because ADL and iADL are typically not available as structured EHR data, our models can enhance researchers’ ability to identify subgroups among people living with dementia according to their ADL and iADL dependency. Our models can improve patient phenotyping and confounding adjustment in EHR data that are used in comparative effectiveness and safety research.

## Appendix

Multimedia Appendix 1 ADL-and iADL-related key term categories, ADL model parameters, iADL model parameters, excerpts of NLP-identified patient notes with evidence of ADL/iADL impairment, prevalence of ADL term groups across positive-labeled cases of ADL impairment in the unfiltered data set, and prevalence of iADL term groups across positive-labeled cases of iADL impairment in the unfiltered data set.

## Abbreviations

ADL: activity of daily living
AUPRC: area under the precision-recall curve
AUROC: area under the receiver operating characteristic curve
BERT: bidirectional encoder representations from transformers
EHR: electronic health record
iADL: instrumental activity of daily living
LASSO: least absolute shrinkage and selection operator
MGB: Mass General Brigham
MIMIC: Medical Information Mart for Intensive Care
NLP: natural language processing
ROC: receiver operating characteristic
RPDR: Research Patient Data Repository
SVM: support vector machine

## Data Availability

The data sets generated and analyzed during this study are not publicly available as they contain protected health information from patient health records.

## References

[1] 2023 Alzheimer's disease facts and figures. The Alzheimer’s Association.

[2] Edemekong PF, Bomgaars DL, Sukumaran S, Caroline C (2023). Activities of Daily Living.

[3] Hung CH, Hung GU, Wei CY, Tzeng RC, Chiu PY (2021). Function-based dementia severity assessment for vascular cognitive impairment. J Formos Med Assoc.

[4] Low DM, Rumker L, Talkar T, Torous J, Cecchi G, Ghosh SS (2020). Natural language processing reveals vulnerable mental health support groups and heightened health anxiety on reddit during COVID-19: observational study. J Med Internet Res.

[5] Savova GK, Danciu I, Alamudun F, Miller T, Lin C, Bitterman DS, Tourassi G, Warner JL (2019). Use of natural language processing to extract clinical cancer phenotypes from electronic medical records. Cancer Res.

[6] Nalichowski R, Keogh D, Chueh HC, Murphy SN (2006). Calculating the benefits of a research patient data repository. AMIA Annu Symp Proc.

[7] Lin KJ, Glynn RJ, Singer DE, Murphy SN, Lii J, Schneeweiss S (2018). Out-of-system care and recording of patient characteristics critical for comparative effectiveness research. Epidemiology.

[8] Lin KJ, Rosenthal GE, Murphy SN, Mandl KD, Jin Y, Glynn RJ, Schneeweiss S (2020). External validation of an algorithm to identify patients with high data-completeness in electronic health records for comparative effectiveness research. Clin Epidemiol.

[9] Lin KJ, Singer DE, Glynn RJ, Blackley S, Zhou L, Liu J, Dube G, Oertel LB, Schneeweiss S (2017). Prediction score for anticoagulation control quality among older adults. J Am Heart Assoc.

[10] Wilkinson T, Ly A, Schnier C, Rannikmäe K, Bush K, Brayne C, Quinn TJ, Sudlow CLM (2018). Identifying dementia cases with routinely collected health data: a systematic review. Alzheimers Dement.

[11] Zhou L, Plasek JM, Mahoney LM, Karipineni N, Chang F, Yan X, Chang F, Dimaggio D, Goldman DS, Rocha RA (2011). Using Medical Text Extraction, Reasoning and Mapping System (MTERMS) to process medication information in outpatient clinical notes. AMIA Annu Symp Proc.

[12] Tibshirani R (2018). Regression shrinkage and selection via the lasso. J R Stat Soc Ser B Methodol.

[13] Breiman L (2001). Random forests. Mach Learn.

[14] Pedregosa F, Varoquaux G, Gramfort A, Michel V, Thirion B, Grisel O, Blondel M, Prettenhofer P, Weiss R, Dubourg V, Vanderplas J, Passos A, Cournapeau D (2011). Scikit-learn: machine learning in Python. J Mach Learn Res.

[15] Chen T, Guestrin C (2016). XGBoost: a scalable tree boosting system. Proceedings of the 22nd ACM SIGKDD International Conference on Knowledge Discovery and Data Mining.

[16] Tf-idf weighting. The Stanford Natural Language Processing Group.

[17] Yang J, Wang L, Phadke NA, Wickner PG, Mancini CM, Blumenthal KG, Zhou L (2020). Development and validation of a deep learning model for detection of allergic reactions using safety event reports across hospitals. JAMA Netw Open.

[18] Alsentzer E, Murphy J, Boag W, Weng WH, Jindi D, Naumann T, McDermott M Publicly available clinical BERT embeddings. ArXive.

[19] Lee J, Yoon W, Kim S, Kim D, Kim S, So CH, Kang J (2020). BioBERT: a pre-trained biomedical language representation model for biomedical text mining. Bioinformatics.

[20] Johnson AEW, Pollard TJ, Shen L, Lehman LWH, Feng M, Ghassemi M, Moody B, Szolovits P, Celi LA, Mark RG (2016). MIMIC-III, a freely accessible critical care database. Sci Data.

[21] Wolf T, Debut L, Sanh V, Chaumond J, Delangue C, Moi A, Cistac P, Rault T, Louf R, Funtowicz M, Davison J, Shleifer S, von Platen P, Ma C, Jernite Y, Plu J, Xu C, Le Scao T, Gugger S, Drame M, Lhoest Q, Rush A HuggingFace's transformers: state-of-the-art natural language processing. ArXive.

[22] Jekel K, Damian M, Wattmo C, Hausner L, Bullock R, Connelly PJ, Dubois B, Eriksdotter M, Ewers M, Graessel E, Kramberger MG, Law E, Mecocci P, Molinuevo JL, Nygård L, Olde-Rikkert MG, Orgogozo J, Pasquier F, Peres K, Salmon E, Sikkes SA, Sobow T, Spiegel R, Tsolaki M, Winblad B, Frölich L (2015). Mild cognitive impairment and deficits in instrumental activities of daily living: a systematic review. Alzheimers Res Ther.

[23] Liu KPY, Chan CCH, Chu MML, Ng TYL, Chu LW, Hui FSL, Yuen HK, Fisher AG (2007). Activities of daily living performance in dementia. Acta Neurol Scand.

[24] Zurlo A, Zuliani G (2018). Management of care transition and hospital discharge. Aging Clin Exp Res.

[25] Salvà A, Roqué M, Rojano X, Inzitari M, Andrieu S, Schiffrin EJ, Guigoz Y, Vellas B (2012). Falls and risk factors for falls in community-dwelling adults with dementia (NutriAlz trial). Alzheimer Dis Assoc Disord.

[26] Schiltz NK, Dolansky MA, Warner DF, Stange KC, Gravenstein S, Koroukian SM (2020). Impact of instrumental activities of daily living limitations on hospital readmission: an observational study using machine learning. J Gen Intern Med.

[27] Kharrazi H, Anzaldi LJ, Hernandez L, Davison A, Boyd CM, Leff B, Kimura J, Weiner JP (2018). The value of unstructured electronic health record data in geriatric syndrome case identification. J Am Geriatr Soc.

[28] Anzaldi LJ, Davison A, Boyd CM, Leff B, Kharrazi H (2017). Comparing clinician descriptions of frailty and geriatric syndromes using electronic health records: a retrospective cohort study. BMC Geriatr.

[29] Lin KJ, Schneeweiss S (2016). Considerations for the analysis of longitudinal electronic health records linked to claims data to study the effectiveness and safety of drugs. Clin Pharmacol Ther.

[30] Sheehan CM, Tucker-Drob EM (2019). Gendered expectations distort male-female differences in instrumental activities of daily living in later adulthood. J Gerontol B Psychol Sci Soc Sci.

[31] Jang SN, Kawachi I (2019). Why do older Korean adults respond differently to activities of daily living and instrumental activities of daily living? A differential item functioning analysis. Ann Geriatr Med Res.

