# Supplementary material for: Identifying Functional Status Impairment in People Living With Dementia Through Natural Language Processing of Clinical Documents: Cross-Sectional Study
Source: J Med Internet Res. 2024 Feb 13;26:e47739. doi: 10.2196/47739 (PMC10900085; doi:10.2196/47739)
Supplement: Multimedia Appendix 1 [file jmir_v26i1e47739_app1.docx]

**Table S1.** ADL- and iADL-related key term categories.

| **ADL** | **iADL** | **Other** |
| --- | --- | --- |
| Activities of Daily Living - General | Instrumental Activities of Daily Living - General | Dementia Severity |
| Grooming | Shopping | Hospice |
| Hygiene | Housekeeping |  |
| Bathing | Driving |  |
| Dressing | Public Transportation |  |
| Feeding | Finances |  |
| Ambulating | Cooking |  |
| Toileting | Medication Management |  |
| Incontinence | Telephone Use |  |

**Table S2.** ADL model parameters.

Logistic Regression

| **Parameter** | **Value** |
| --- | --- |
| Max iterations | 1000 |
| Learning rate | Adaptive |
| Eta0 (initial learning rate) | 0.5 |

LASSO

| **Parameter** | **Value** |
| --- | --- |
| Max iterations | 2000 |
| Learning rate | Adaptive |
| Eta0 (initial learning rate) | 0.3 |

SVM

| **Parameter** | **Value** |
| --- | --- |
| Max iterations | 2000 |
| Learning rate | Adaptive |
| Eta0 (initial learning rate) | 0.1 |

Random Forest

| **Parameter** | **Value** |
| --- | --- |
| N estimators | 300 |
| Max depth | None |

XGBoost

| **Parameter** | **Value** |
| --- | --- |
| Eta (learning rate) | 0.5 |
| Max depth | 100 |
| Gamma (min split loss) | 0 |

Deep Learning

| **Parameter** | **Value** |
| --- | --- |
| Word embedding dimensions | 100 |
| Character embedding dimensions | 30 |
| Min word frequency cutoff | 5 |
| LSTM hidden layers | 1 |
| LSTM hidden layer size | 200 |
| CNN hidden layers | 4 |
| CNN hidden layer size | 50 |
| Dropout | 0.5 |
| Optimizer | SGD |
| Batch size | 10 |
| Learning rate | 0.2 |
| Epochs (iterations) | 20 |

Bio+Clinical BERT

| **Parameter** | **Value** |
| --- | --- |
| Batch size | 8 |
| Learning rate | 3e-5 |
| Weight decay | 0.01 |
| Epochs (iterations) | 3 |

**Table S3.** iADL model parameters.

Logistic Regression

| **Parameter** | **Value** |
| --- | --- |
| Max iterations | 1000 |
| Learning rate | Adaptive |
| Eta0 (initial learning rate) | 0.5 |

LASSO

| **Parameter** | **Value** |
| --- | --- |
| Max iterations | 1000 |
| Learning rate | Optimal |
| Eta0 (initial learning rate) | N/A |

SVM

| **Parameter** | **Value** |
| --- | --- |
| Max iterations | 1000 |
| Learning rate | Adaptive |
| Eta0 (initial learning rate) | 0.3 |

Random Forest

| **Parameter** | **Value** |
| --- | --- |
| N estimators | 300 |
| Max depth | None |

XGBoost

| **Parameter** | **Value** |
| --- | --- |
| Eta (learning rate) | 0.5 |
| Max depth | 100 |
| Gamma (min split loss) | 0 |

Deep Learning

| **Parameter** | **Value** |
| --- | --- |
| Word embedding dimensions | 100 |
| Character embedding dimensions | 30 |
| Min word frequency cutoff | 3 |
| LSTM hidden layers | 1 |
| LSTM hidden layer size | 200 |
| CNN hidden layers | 4 |
| CNN hidden layer size | 50 |
| Dropout | 0.5 |
| Optimizer | SGD |
| Batch size | 10 |
| Learning rate | 0.5 |
| Epochs (iterations) | 30 |

Bio+Clinical BERT

| **Parameter** | **Value** |
| --- | --- |
| Batch size | 8 |
| Learning rate | 3e-5 |
| Weight decay | 0.01 |
| Epochs (iterations) | 3 |

**Table S4.** Excerpts of NLP-identified patient notes with evidence (in bold) of ADL/iADL impairment.

| ADL | pt classified w/ nonspecific protein **calorie malnutrition** given increased metabolic stress r/t thoracic surgery and inadequate TF infusion since surgery. |
| --- | --- |
|  | supervision **assistance with feeding** - slow rate, allow pt plenty of time to initiate complete swallow. |
|  | he is min **assist for grooming** with mod cues, min **assist for toilet transfers**. |
| iADL | has **assistance for** bathing, dressing, **medication and meal prep**. |
|  | claims **girlfriend does his iADLs such as cooking**. |
|  | has home **health aide 2 days week for shopping, transportation**. |
|  | |

**Table S5.** Prevalence of ADL term groups across positive-labeled cases of ADL impairment in the unfiltered dataset.

| **Group** | **Prevalence** |
| --- | --- |
| Ambulating | 1.1% |
| Activities of Daily Living - General | 0.5% |
| Toileting | 0.2% |
| Bathing | 0.2% |
| Incontinence | 0.1% |
| Feeding | 0.1% |
|  | |

**Table S6.** Prevalence of iADL term groups across positive-labeled cases of iADL impairment in the unfiltered dataset.

| **Group** | **Prevalence** |
| --- | --- |
| Finances | 0.3% |
| Medication management | 0.1% |
| Cooking | 0.1% |
| Shopping | 0.1% |
| Housekeeping | 0.1% |
| Instrumental Activities of Daily Living - General | 0.1% |
|  | |
